# Integrated Online-to-Offline Model of Care for HIV Prevention and Treatment Among Men Who Have Sex With Men in Malaysia: Protocol for an Intervention Development and a Multiphase Trial

**DOI:** 10.2196/60962

**Published:** 2024-10-23

**Authors:** Antoine Khati, Jeffrey A Wickersham, Kamal Gautam, Kiran Paudel, Panyaphon Phiphatkhunarnon, Sin How Lim, Kirthana Puniamurthy, Frederick L Altice, Nittaya Phanuphak, Iskandar Azwa, Roman Shrestha

**Affiliations:** 1 Department of Allied Health Sciences University of Connecticut Storrs Mansfield, CT United States; 2 Section of Infectious Diseases Department of Internal Medicine Yale School of Medicine New Haven, CT United States; 3 Love Foundation Chiang Mai Thailand; 4 Centre of Excellence for Research in AIDS, Faculty of Medicine, University of Malaya Kuala Lumpur Malaysia; 5 Institute of HIV Research and Innovation Bangkok Thailand

**Keywords:** HIV, HIV testing, pre-exposure prophylaxis, antiretroviral therapy, CINTAI, men who have sex with men, online-to-offline model, eHealth

## Abstract

**Background:**

HIV continues to have a disproportionate impact on specific populations in Malaysia, particularly men who have sex with men (MSM). HIV self-testing (HIVST) is a strategy that has been shown to scale up HIV testing rates. However, it faces shortcomings because of concerns about self-efficacy, result interpretation, and lack of counseling and linkage to care. This underscores the need for an innovative approach that integrates HIVST with timely counseling, expert guidance, and referrals to enhance engagement in relevant HIV prevention or treatment.

**Objective:**

This study aims to describe the protocol used in developing and testing a web-based platform (ie, CINTAI) providing an HIVST kit and real-time e-counseling to support online-to-offline linkage to HIV care services for MSM in Malaysia.

**Methods:**

The methods are reported according to the SPIRIT (Standard Protocol Items: Recommendations for Interventional Trials) 2013 guidelines. In phase I, we will adapt existing HIVST web-based platforms to create a new online-to-offline HIVST and counseling platform called “CINTAI” for Malaysian MSM. In phase II, we will use a type 1 hybrid implementation trial design to determine the feasibility, acceptability, and preliminary efficacy of “CINTAI” compared with treatment as usual among Malaysian MSM, with assessments conducted over 6 months. Multilevel implementation factors will also be collected to guide future adoption and scale-up. We will enroll 78 MSM in the pilot randomized controlled trial. Baseline characteristics will be tested for homogeneity between groups using appropriate statistical tests. A generalized linear mixed model with random subject effects will account for within-subject correlation. Treatment assignment, time, interaction, and confounders will be included. The proportion of MSM tested for HIV over 6 months and other outcomes (pre-exposure prophylaxis for HIV or antiretroviral therapy linkage, HIV risk behaviors, and chemsex harm reduction) will be compared using linear contrasts.

**Results:**

We completed phase I of the proposed study in April 2024 and started phase II in May 2024, with 15 participants recruited (7 in the CINTAI and 8 in the treatment-as-usual groups). On the basis of a series of formative works completed during phase I, we developed a fully functional, web-based platform that provides a digital platform for MSM in Malaysia to order HIVST kits for free and to receive HIV counseling, followed by offline linkage to HIV prevention services (if HIV negative) or HIV treatment services (if HIV positive).

**Conclusions:**

Despite being at high risk for HIV transmission, MSM in Malaysia have alarmingly low testing and linkage to HIV care services, prompting the need for innovative approaches to support HIV prevention efforts. If found to be feasible and acceptable, CINTAI can be easily adapted for a range of health outcomes and health care delivery services for MSM, including adaptation to other low- and middle-income countries.

**International Registered Report Identifier (IRRID):**

DERR1-10.2196/60962

## Introduction

As opposed to overall declining HIV trends globally, its incidence is rising among certain vulnerable subpopulations, such as sexual and gender minority groups, and in some regions of the world, particularly in Southeast Asia [1-3]. Men who have sex with men (MSM) are one such example and constitute a disproportionate fraction of people living with HIV [1,3]. In Malaysia, a country in the Asia-Pacific region, the HIV epidemic has transitioned to MSM and led to climbing mortality rates within the past decade [4,5]. Several contributing factors have exacerbated the HIV epidemic in Malaysia among MSM. Malaysian MSM are at a disproportionate risk for comorbid substance use and psychiatric disorders, which also fuel transmission, including depression and amphetamine-type stimulant use, which has led, in recent years, to an increase in sexualized drug use (“chemsex”) in this subgroup, further compounding risky behaviors such as condomless sex and multiple sexual partners [6-10]. Therefore, there is a greater urgency for routine HIV testing to expedite prevention, early diagnosis, and treatment.

Although HIV testing guidelines indicate that MSM with high-risk sexual behaviors (eg, condomless receptive anal sex and multiple anal sex partners) are recommended to receive testing every 3-6 months [11], HIV testing rates among MSM in Malaysia remain alarmingly low according to recent studies, with 33.8% of them never having tested for HIV in the past and only around 30% having tested for HIV in the past 6 months [12]. Low HIV testing uptake and linkage to care in this subgroup can be explained by several factors, including the criminalization of same-sex sexual activity and ensuing stigmatization around HIV, compounded by fears around disclosing sexuality and prevailing distrust of health care professionals, which render access to care more complex and suboptimal [13-16].

eHealth, defined as the use of information and communication technologies in health care delivery, dismantles social access barriers to care and, therefore, has the potential to improve accessibility to services among stigmatized populations, especially in Malaysia [17-20]. The stigma- and discrimination-conducive setting in Malaysia renders Malaysian MSM prime potential beneficiaries of such a strategy, coupled with their widespread use of web technologies for sexual health [21]. As such, Malaysian MSM who are reluctant to “come out” may find it a more accessible and less stigmatizing method of obtaining resources and care.

HIV self-testing (HIVST) is an effective strategy to maximize confidentiality while avoiding conventional HIV testing facilities. However, its use in Malaysia remains low (25.2%) due to a lack of self-efficacy for HIVST, concerns about misinterpreting results, and missed opportunities for counseling and linkage to care [22]. The median out-of-pocket health expenditure for patients with HIV/AIDS in Malaysia was RM 1080 (US $254.48) per year, representing 14.7% of patients’ median income [23]. This indicates the need for an innovative approach meant to complement HIVST with access to timely HIV knowledge and expertise from clinicians, as well as guidance on using and interpreting the result and recommended referrals or next steps.

To fill this gap, we aim to develop a web-based HIVST kit delivery platform (ie, CINTAI; “love” in Bahasa Malaysia) designed for MSM in Malaysia that provides free and anonymous HIVST kits as well as real-time e-counseling for HIVST (eHIVST) integrated with online-to-offline (O2O) linkage to HIV and other harm reduction support services. Guided by implementation science and a commitment to health equity, the study seeks to generate robust ideas on the feasibility and acceptability of this intervention for potential adaptation in other low- and middle-income countries. This paper aims to describe the protocol that will be used in the development and pilot testing of “CINTAI” to improve HIV testing and linkage to HIV prevention and treatment services among MSM in Malaysia. The implementation of web-based interventions like CINTAI will significantly increase the rates of HIV testing and linkage to HIV prevention and treatment services among MSM in Malaysia compared with the current standard of care.

## Methods

### Multiphase Study Design

The methods are reported according to the SPIRIT (Standard Protocol Items: Recommendations for Interventional Trials) 2013 guidelines [24]. This study will be conducted in 2 phases. In phase I (development phase), we will adapt existing HIVST web-based platforms to create a new O2O HIVST and counseling platform called “CINTAI” for Malaysian MSM. In phase II (testing phase), we will use a type 1 hybrid implementation trial design to determine the feasibility, acceptability, and preliminary efficacy of the CINTAI platform. Additionally, we will assess multilevel implementation factors using the Consolidated Framework for Implementation Research (CFIR) to guide future adoption and scale-up of the CINTAI platform.

### Theoretical Framework

The Behavioral Model for Vulnerable Populations will guide intervention development and implementation [25]. This model indicates that health behavior can be explained in terms of predisposing factors (eg, characteristics of the individuals or community that influence health service use), enabling factors (eg, personal, family, and community resources), and need factors (eg, perceived and evaluated health status). This was chosen as the model for this study because it incorporates specific vulnerabilities and competing needs that lead to significant barriers to obtaining health care among Malaysian MSM, and it has been used in a wide variety of geographical contexts and patient populations.

### Phase I: Development of the CINTAI Platform

#### Overview

**Figure 1:**
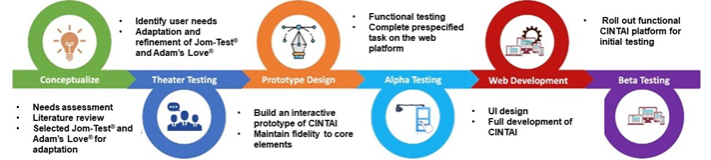
Description of study procedural phases. UI: user interface.

We will adapt and refine the existing platforms (ie, Jom-Test and Adam’s Love) using the modified Intervention Mapping for Adaptation framework to develop the CINTAI platform for optimal use among Malaysian MSM. The modified Intervention Mapping for Adaptation model comprises the sequential steps listed below. The study procedural phases are described in Figure 1.

#### Jom-Test and Adam’s Love

Adam’s Love is a web-based O2O HIV service delivery platform designed to improve HIV testing and pre-exposure prophylaxis for HIV (PrEP) uptake among MSM in Thailand. Its key features included risk assessment, real-time e-counseling (instant messaging, SMS text messaging, and audio or video call), an online site- and service-specific booking system, and a real-time monitoring feature to track and monitor individuals’ progress. It has demonstrated success in engaging hard-to-reach Thai MSM in e-counseling, assessing risks in real time, and effectively linking them online to relevant clinical services using its novel O2O model [26]. Jom-Test, on the other hand, is a web-based HIVST platform developed to promote HIV testing by providing free and anonymous HIVST kits to MSM [27].

#### Phase IA: Theater Testing

##### Participants and Recruitment

We will conduct focus group discussions (FGDs) with MSM and stakeholders to adapt, expand, and refine the content and functionalities of Jom-Test and Adam’s Love to create an interactive prototype of the CINTAI platform. We aim to conduct 6 FGDs with 6-7 MSM per group and 3 FGDs with 6-7 stakeholders per group to theater test the existing platform to develop an interactive prototype of CINTAI. We will continue to recruit participants until saturation is achieved. Eligibility criteria for MSM will include identifying as MSM (HIV negative, HIV positive, or HIV status unknown) and being 18 years of age or older. Clinical stakeholders will include counselors, nongovernmental organization and clinic staff, and physicians who provide HIV-related services to MSM in Malaysia. MSM living with HIV are included in the eligibility criteria to learn their unique perspectives in navigating through the Malaysian health system and accessing HIV treatment services. We will recruit MSM participants using posts and social media advertisements, geospatial dating apps (eg, Grindr and Hornet), peer referrals, and announcements in MSM-led or serving community organizations.

##### Theater Testing Procedures

Participants will first be screened using an online questionnaire to assess their eligibility. We will then conduct the FGDs in person using 2 semistructured interview guides (for MSM or clinical stakeholders). FGDs will be audio recorded and transcribed for later analysis. If the in-person FGD does not provide sufficient and meaningful information or if many potential participants are reluctant to participate in an in-person session, we will offer an option to participate in a remote FGD session using videoconferencing technology.

##### Session Content

The FGDs among MSM will broadly explore the 3 domains of the Behavioral Model for Vulnerable Populations framework [25] related to the target population, including *predisposing factors* (demographic, substance use, and mental health), *enabling resources* (income, transportation, available clinical and support services, and discrimination in the health care setting), and *need factors* (perceived health status). Sessions for both MSM and clinical stakeholders will involve feedback regarding how the digital platform could improve HIV testing frequency and linkage to prevention and treatment services. Sessions will also foster discussion by addressing limitations found in existing platforms, including additional features unique to the Malaysian context, such as e-counseling with O2O service delivery and integration of low-threshold harm reduction services.

#### Phase IB: Alpha Testing

In this step, the results from the theater testing phase will be used to refine and improve the content and functions of the existing platforms and tailor them to the Malaysian MSM. This will lead to the development of an interactive prototype of CINTAI.

##### Participants and Recruitment

Screening and enrollment of MSM (n=5; HIV negative or HIV status unknown only) and clinical stakeholders (n=5) will be identical to methods used for the theater testing.

##### Procedures

Individual one-on-one “thinking aloud” sessions [28] will occur between the participant and a facilitator. Each session will be video recorded to capture the participant’s experience with the “CINTAI” prototype. Participants will be asked to complete specific tasks, including profile setup, risk assessment, HIVST kit and PartyPack [29] ordering, scheduling of e-counseling sessions for HIV testing or chemsex, and finding PrEP providers using the locator feature, all while narrating their thoughts. After this is completed, participants will fill out a questionnaire assessing platform testing–related experiences as well as their experience completing the given tasks using the prototype. Open-ended questions will be used to assess the ease and experience of use, recommended modifications, and overall feasibility. Clinical stakeholders will provide specific feedback regarding the clarity, usefulness, and format of the visual summary of user data.

##### Analysis

Recorded sessions will be transcribed, and facilitator notes will be compiled to examine and identify usability problems. Verbal indicators of usability problems from participants, such as doubt, task difficulty, incomprehensibility, or annoyance, will be scanned, and the total number of problems will be collated. A distinction will be made according to how issues surfaced in the data: observation of behavioral data, verbalization by the participants, or both. Based on these results, recommendations for further modifications will be synthesized to prepare for the beta version (phase IC).

##### Expected Elements of the CINTAI Platform (Beta Version)

**Figure 2:**
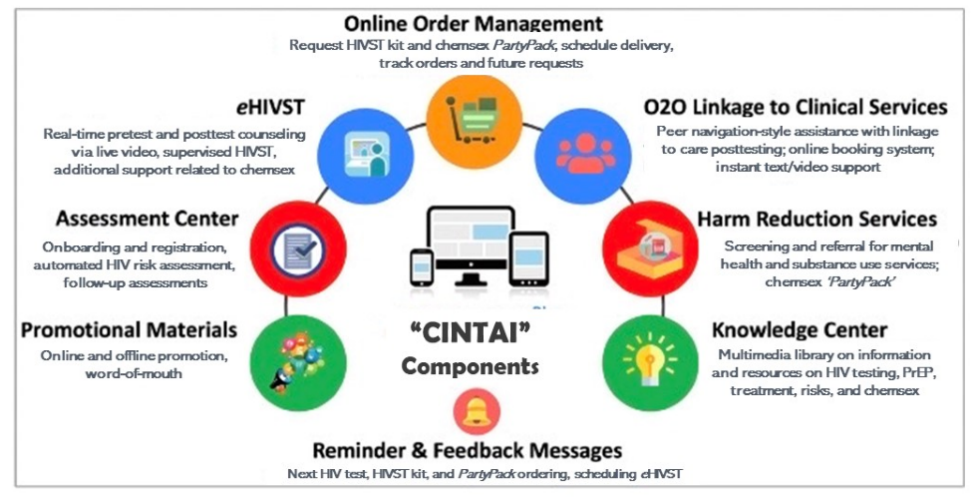
Features in the CINTAI digital platform. HIVST: HIV self-testing; O2O: online-to-offline; PrEP: pre-exposure prophylaxis for HIV.

Elements of “CINTAI” will minimally include features already present in Jom-Test (eg, ordering of an HIVST kit and written instructions for using HIVST) and Adam’s Love (eg, e-counseling support via instant messaging, SMS text messaging, or video or audio call; online booking system; and risk calculator tool), as well as the following additional components: a monitoring dashboard, geocoded location options for HIV testing, HIV prevention and treatment clinics and chemsex-related services, and peer navigation-style assistance, to increase user engagement and provide a seamless handoff to clinical services. Additionally, the platform will incorporate screening of engagement in chemsex as well as real-time e-counseling on chemsex harm reduction strategies, including referrals to suitable care and support services. The platform will also include a chemsex harm reduction kit called the “PartyPack.” The PartyPack is a feasible and acceptable HIV and sexually transmitted infection prevention tool previously used among MSM in Thailand [29,30] and will be incorporated in “CINTAI” as an optional “add-on” that users can request. The PartyPack is a small box containing condoms, personal lubricant, and a QR code that, when scanned through a smartphone, links users to chemsex harm reduction resources on the “CINTAI” platform. This will ensure that individuals who intend to engage in chemsex have access to the information and tools necessary to lower their health risks in a nonjudgmental environment. Features of the “CINTAI” platform are depicted in Figure 2.

#### Phase IC: Beta Testing

Beta testing is conducted to identify any remaining bugs or usability issues of the newly developed platform in a “real world” setting. This period will last 2 months and will be conducted to finalize the software’s design, functionality, and usability.

##### Participants and Recruitment

Recruitment and screening of participants will be identical to prior phases of the project. A sample size of 10 participants was chosen based on recommendations for initial beta testing of a new technology [31]. Eligible participants will be at least 18 years old; HIV negative or of unknown HIV status; cisgender men; identify as MSM; and have access to a smartphone, computer, or internet-enabled tablet [31].

##### Procedures

Participants will be asked to complete a pre-use survey that will collect sociodemographic characteristics as well as information about perceived barriers to HIV testing access, and linkage to HIV prevention and treatment for services uptake. After completing the survey, participants will attend the CINTAI onboarding tutorial and access the platform using a single-use registration access code. Participants will be asked to use the CINTAI platform for 60 days and encouraged to use all its components. On day 60, participants will be asked to complete a post-use survey using the Systems Usability Scale (SUS), which will take around 20-30 minutes [32]. To evaluate engagement and interest, web analytics will be collected, including the number of log-ins, the duration of each session, pages visited, and features used on the CINTAI platform. Lastly, audio-recorded, exit, one-on-one interview sessions will be conducted to explore participant feedback on the functionality of the platform, including the errors encountered, and their motivation to use CINTAI to inform further refinement of the platform. One-to-one interviews can last from 30 to 60 minutes, depending on the participants’ nature.

##### Analysis

The score of the SUS usability tool [32] ranges from 0 to 100, and a cutoff of 50 is the industry standard for acceptance usability [33], but we will be using a score of ≥70 as being minimally acceptable. Differences in pre- or postuse surveys will be analyzed using 2-tailed *t* tests or Wilcoxon rank-sum tests to evaluate changes in HIV testing and subsequent linkage to clinical services. The reliability of the scale will be measured by calculating Cronbach α. Qualitative data will be analyzed using the same approach as theater testing FGDs.

### Phase II: Testing of the CINTAI Platform

#### Overview

We will conduct a hybrid type 1 implementation science trial [34] that involves an assessment of the feasibility, acceptability, and preliminary efficacy of the CINTAI platform, while simultaneously assessing contextual implementation factors to guide its future adoption and scale-up. The hybrid type 1 efficacy trial provides a heuristic to assess implementation factors during (not after) the efficacy study. A summary of phase II study activities and measures is shown in Table 1.

**Table 1:** Study activity and measures (phase II).

Study activity				Timeline						
				Prebaseline		Baseline		3 months		6 months
**Enrollment**										
	Eligibility screen			✓						
	Informed consent					✓				
	Randomization					✓				
**Interventions**										
	Website onboarding					✓				
	Access to the website									
		CINTAI group			✓		✓		✓	
		TAU^a^ group			✓		✓		✓	
**Assessments**										
	Focus groups					✓		✓		✓
	**Feasibility and acceptability**									
		Feasibility of screening^b^					✓		✓	
		HIVST^c^ management^d^					✓		✓	
		eHIVST^e^ utilization^f^					✓		✓	
		Usability^g^					✓		✓	
		Web analytics					✓		✓	
	**Preliminary efficacy**									
		HIV testing					✓		✓	
		Linkage to PrEP^h^ or ART^i^					✓		✓	
		Engagement in HIV risky behavior					✓		✓	
		Uptake of harm reduction services^j^					✓		✓	
Payment						✓		✓		✓

^a^TAU: treatment as usual.

^b^Feasibility of screening, recruitment, randomization, retention, and fidelity to study protocol.

^c^HIVST: HIV self-testing.

^d^HIVST kit and PartyPack distribution data retrieved from the HIVST management dashboard.

^e^eHIVST: e-counseling for HIVST.

^f^Use of eHIVST and chemsex-related services (screening and e-counseling).

^g^Subjective usability of CINTAI using the System Usability Scale.

^h^PrEP: pre-exposure prophylaxis for HIV.

^i^ART: antiretroviral therapy.

^j^Uptake of chemsex-related harm reduction services: mental health counselors, psychiatrists, and needle exchange programs.

#### Phase IIA: Pilot Trial of the CINTAI Platform

##### Study Design

**Figure 3:**
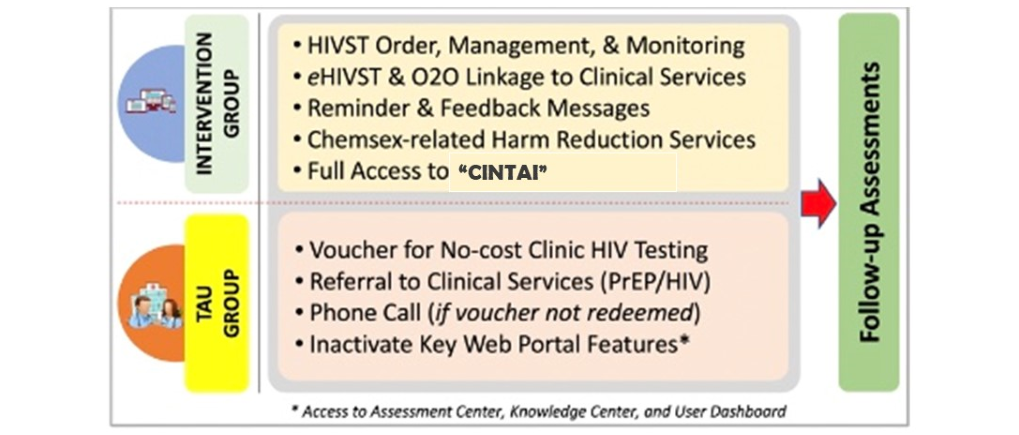
Study design of the CINTAI trial (phase IIA). eHIVST: e-counseling for HIV self-testing; HIVST: HIV self-testing kits; O2O: online-to-offline; TAU: treatment as usual.

We will conduct a prospective randomized controlled trial to evaluate the CINTAI platform versus treatment as usual (TAU) among MSM in Malaysia for primary (ie, feasibility and acceptability) and secondary (ie, preliminary efficacy) outcomes over 6 months of observation, with assessments at 3 and 6 months. We will enroll 78 participants who will be randomized (1:1) to receive either CINTAI or TAU to ensure a balance between study arms [35,36] (Figure 3).

##### Participants, Recruitment, and Procedures

Eligibility criteria and recruitment measures will be identical to procedures used for the beta testing period (phase IC). Potential participants will fill out a web-based screening form to assess eligibility. Eligible participants will receive a call from researchers where they will be informed about the study and guided through consent procedures. Participants will then complete a baseline assessment and be randomized to CINTAI or TAU. Once randomization is complete, participants will be introduced to the staff members and study procedures.

Participants in the CINTAI group will be given access to all features, including an assessment center, online registration, risk calculator, HIVST kit order, management and monitoring, automated reminders, real-time e-counseling, chemsex-related harm reduction services, chat functions, and a knowledge center. These participants will follow a predetermined pathway to accessing services such as HIVST kit ordering, e-counseling, and subsequent O2O linkage to HIV clinical services. The TAU group participants will be given access to the CINTAI platform, with major intervention features being deactivated. The TAU group participants can only access the assessment center, knowledge center, and a voucher for no-cost community-based HIV testing. Participants in both groups will be followed for 6 months, with assessments at 3 and 6 months, which will take around 20-30 minutes to complete.

##### Primary Outcomes

Feasibility will be measured using metrics for the number of individuals screened, enrolled, and randomized. Retention will be monitored using web analytics showing the frequency and length of “CINTAI” use. Acceptability will be measured using the SUS usability tool (mentioned above; mean score of ≥70 for minimum acceptability) [32,37], HIVST kit and PartyPack [29] distribution, use of O2O e-counseling, and analysis of qualitative data from FGDs. In the previous study, the Cronbach α value of SUS was 0.95 [38].

##### Secondary Outcome

Preliminary efficacy will be assessed through several constructs. First, counselors will document the completion of HIV testing at the defined intervals (months 3 and 6) in the study database. To assess possible testing events occurring outside the study, participants will be asked whether they received any additional HIV testing as well as extract testing events via medical records. Second, posttest linkage to PrEP (HIV–) or antiretroviral therapy (ART; HIV+) will be measured, including the date of the first clinic visit, time to ART or PrEP initiation, and all clinic visit dates for the first 6 months of follow-up (to measure retention in care; self-report and extract from medical records).

##### Analytical Plan

Baseline characteristics will be tested for homogeneity between both groups using the 2-tailed *t* test or the Wilcoxon rank sum test for continuous variables and the *χ*^2^ test or the Fisher exact test for categorical variables. Between-group differences will be adjusted for in the model. A generalized linear mixed model [39] with random subject effects will be built to account for the correlation in repeated measurements that occur within subjects. Treatment assignment, time, the interaction between time and treatment assignment, and any other confounders will be included as covariates. The proportion of MSM who have tested for HIV over the 6-month study period will be estimated and compared using linear contrasts. Similar analyses will be conducted for other outcomes (linkage to PrEP or ART, engagement in HIV risk behaviors, and uptake of chemsex-related harm reduction services).

##### Sample Size

Power analyses conducted in R using the power package (R Foundation for Statistical Computing) for single-level generalized linear models [40] suggest 80% power to detect a statistically significant (*P*<.05) medium effect size (Cohen *d*=0.15) with 64 participants. An additional 14 participants (approximately 20% of the sample size) will be recruited to account for potential attrition, for a total of 78 participants or 39 participants per study condition.

#### Phase IIB: Explore Multilevel Implementation Factors

The CFIR will be used to gather multilevel implementation factors for future adoption. CFIR provides a structured menu of constructs associated with effective implementation and can be used flexibly at any phase [41,42]. It consists of 5 domains with 39 underlying constructs [41]. As recommended [41], we have identified 11 constructs relevant to our trial (Table 2).

**Table 2:** CFIR^a^ constructs to explore multilevel implementation factors.

Domains	Constructs	Sample questionnaires
Intervention	Relative advantage Adaptability Design quality and packaging	How does CINTAI compare with other alternatives? What changes will be needed for CINTAI to work effectively? What is your perception of the O2O^b^ model of service delivery?
Internal context	Structural characteristics Readiness for implementation	What kinds of infrastructure changes will be needed to accommodate the program? What level of endorsement or support have you seen or heard from leaders?
External context	Patient needs and resources	What barriers will the users face while using CINTAI?
Participants	Knowledge and beliefs Self-efficacy	Do you have any feelings of anticipation? Stress? Enthusiasm? Why? How confident do you think your colleagues feel about using CINTAI?
Process	Planning Engaging	What have you done (or what do you plan to do) to implement CINTAI? What steps have been taken to encourage people to use CINTAI?

^a^CFIR: Consolidated Framework for Implementation Research.

^b^O2O: online-to-offline.

We will conduct FGDs with the CINTAI group participants (n=20) and clinical stakeholders (eg, clinicians and administrative staff from nongovernmental organizations, clinics, and hospitals; n=15), where we will measure implementation and process measures, existing and potential barriers and facilitators, as well as identify available resources and key community partners. Screening, enrollment, and FGD procedures will be identical to the procedures used for alpha testing. Participants will be asked questions related to the multilevel implementation factors based on the sample guide available elsewhere [43] (Table 2) but tailored for the Malaysian context. Results are expected to provide insights into the feasibility and acceptability of the platform, including barriers to and facilitators of implementation, and will be used to improve the future adoption and implementation of the CINTAI platform nationwide and in other low- and middle-income countries.

### Ethical Considerations

The institutional review board at the University of Connecticut approved this study’s protocol (H22-0075), with an institutional reliance agreement with the University of Malaya. All staff notes, audiotapes, participant information, and transcripts will be kept in a separate locked cabinet within the offices in Centre of Excellence for Research in AIDS (CERiA) and destroyed once uploaded to the University of Connecticut’s secure server, which is HIPAA (Health Insurance Portability and Accountability Act) compliant. After explaining the details of the study, such as aims, possible risks, and benefits, the research assistants will obtain informed consent from participants. All participants will be reminded that their refusal to participate in no way will negatively affect their relationship with any of the participating agencies or clinics and their ability to continue medical services from area clinics in the future. The principal investigator and all research staff will make every effort to protect the privacy and confidentiality of the participant information. In Phase II, participants will receive 40 RM (approximately US $9.31) at three assessment time points (baseline, 3 months, and 6 months) for their time and effort in participating in the study.

## Results

The implementation of theater testing, alpha testing, and beta testing will result in tangible improvements in the functionality, engagement, and user satisfaction of “CINTAI.” The linkage between site users and their clinical providers will also become more seamless. We completed phase I of the proposed study in April 2024. We started phase II in May 2024, with 15 participants recruited (7 in the CINTAI and 8 in the treatment as usual groups) as of September 2024. We anticipate that participant recruitment and data collection will be completed in early to mid-2025. On the basis of a series of formative works completed during this phase, we have developed a fully functional, Malaysia’s first comprehensive, online HIVST platform with integrated counseling, that is, the CINTAI website. The term “CINTAI” derives from the local language, signifying “Love.” CINTAI provides a digital platform for MSM in Malaysia to facilitate their engagement in HIV prevention in a fast and convenient manner. It offers HIV prevention (ie, HIV testing) and other support services (eg, referral to mental health services). We have also developed a web-based “CINTAI Clinic Dashboard,” which provides role-based access to research staff to facilitate patient care for CINTAI users.

## Discussion

### Principal Findings

MSM are among the most at-risk groups for HIV transmission, yet HIV testing and PrEP usage have remained low in Malaysia. The objective of this study is to develop and test a contextual web-based platform for Malaysian MSM with features of HIVST, real-time e-counseling, and O2O linkage to HIV prevention and treatment.

The CINTAI platform will help break different individual, social, and structural barriers by (1) delivering relevant information and resources to MSM during the critical “window” of testing; (2) educating MSM about HIV risk behaviors, strategies for risk reduction, and supporting for linkage to HIV prevention, treatment, and harm reduction services in the community; (3) empowering MSM by enabling e-counselors to develop strategies for posttest linkage to offline services; (4) decreasing long clinic wait times and reducing the number of clinical visits; and (5) collaborating with local stakeholders to facilitate linkage between eHIVST and subsequent offline clinical services (prevention, treatment, and chemsex-related harm reduction services).

We anticipate that the CINTAI platform will be an optimal intervention in diverse settings, which will be determined later after exploring multilevel implementation factors for future adoptions. If successful, the CINTAI platform will be among the first intervention packages for MSM clients, clinicians, and the overall health system to improve the uptake of HIV prevention and testing in Malaysia.

### Conclusions

Suboptimal access to care among MSM in Malaysia has left them at increased risk for HIV and comorbid substance use and psychiatric disorders. The CINTAI platform has the potential to improve access to HIVST, prevention and treatment, and harm reduction services in this subgroup, providing a seamless route to these services for those at risk. If CINTAI is found to be feasible and acceptable, it can be continuously adapted and improved with the goal of reducing HIV incidence and bolstering access to HIV care among MSM in Malaysia.

## Appendix

Multimedia Appendix 1 Peer-review report by HIV/AIDS Intra- and Inter-personal Determinants and Behavioral Interventions Study Section (HIBI) - Risk, Prevention and Health Behavior Integrated Review - Center for Scientific Review (National Institutes of Health, USA).

## Abbreviations

ART: antiretroviral therapy
CERiA: Centre of Excellence for Research in AIDS
CFIR: Consolidated Framework for Implementation Research
eHIVST: e-counseling for HIV self-testing
FGD: focus group discussion
HIPAA: Health Insurance Portability and Accountability Act
HIVST: HIV self-testing
MSM: men who have sex with men
O2O: online-to-offline
PrEP: pre-exposure prophylaxis for HIV
SPIRIT: Standard Protocol Items: Recommendations for Interventional Trials
SUS: Systems Usability Scale
TAU: treatment as usual
