# Supplementary material for: Integrated Online-to-Offline Model of Care for HIV Prevention and Treatment Among Men Who Have Sex With Men in Malaysia: Protocol for an Intervention Development and a Multiphase Trial
Source: JMIR Res Protoc. 2024 Oct 23;13:e60962. doi: 10.2196/60962 (PMC11541154; doi:10.2196/60962)
Supplement: Multimedia Appendix 1 [file resprot_v13i1e60962_app1.pdf]

SUMMARY STATEMENT
(Privileged Communication)

Release Date:	04/01/2022
Revised Date:	
Application Number:	1 R34 MH130233-01A1
Formerly:	1R34MH130233-01

Principal Investigators (Listed Alphabetically):

SHRESTHA, ROMAN (Contact)
WICKERSHAM, JEFFREY ALLEN

Applicant Organization: UNIVERSITY OF CONNECTICUT STORRS

Review Group: HIBI
HIV/AIDS Intra- and Inter-personal Determinants and Behavioral Interventions Study Section
AIDS - EXP. REV.

Meeting Date:	03/10/2022	*RFA/PA:*	PA20-141
Council:	MAY 2022	*PCC:*	9A-ASPT
Requested Start:	07/01/2022		

Project Title:	Integrated online-to-offline (O2O) model of care for HIV prevention and treatment among men who have sex with men
SRG Action:	Impact Score:20 Percentile:4 +
Next Steps:	Visit https://grants.nih.gov/grants/next_steps.htm
Human Subjects:	30-Human subjects involved - Certified, no SRG concerns
Animal Subjects:	10-No live vertebrate animals involved for competing appl.
Gender:	1A-Both genders, scientifically acceptable
Minority:	5A-Only foreign subjects, scientifically acceptable
Age:	3A-No children included, scientifically acceptable

ADMINISTRATIVE BUDGET NOTE: The budget shown is the requested budget and has not been adjusted to reflect any recommendations made by reviewers. If an award is planned, the costs will be calculated by Institute grants management staff based on the recommendations outlined below in the COMMITTEE BUDGET RECOMMENDATIONS section.

1R34MH130233-01A1 Shrestha, Roman

RESUME AND SUMMARY OF DISCUSSION: This application proposes to enhance the previously developed Jom-Test® platform, an anonymous, web-based HIV self-testing platform for MSM in Malaysia, by adding real-time e-counseling and an online-to-offline linkage to HIV prevention and treatment. First, theater testing will be conducted with MSM and stakeholders to develop the interactive form of Jom-Test®, which will be called Jom-TestPlus. A prototype will be then be alpha tested. After further development, the Jom-TestPlus platform will be beta tested for usability and acceptability. Finally, a Hybrid Type-1 implementation science trial will be conducted with 78 Malaysian MSM to assess the acceptability, feasibility and preliminary efficacy of the Jom-TestPlus platform compared to treatment-as-usual. The prevalence of HIV among MSM in Malaysia is very high and the strong, experienced team presents evidence that sexualized drug use (chemsex) contributes to high-risk sexual practices among Malaysian MSM. The committee found this resubmission to be highly responsive to the many concerns that were raised in the prior review of this application. The addition of Drs. Lim and Yee to the team and the inclusion of a consultant – the PI of the Adam's Love study – significantly strengthened the team. A theoretical model, the Behavioral Model for Vulnerable Populations, is now used to direct the analyses and the qualitative data plans have been revised. Additional details were provided to address concerns about pre- and post-test counseling and the need to focus on online-to-offline linkages. Many other concerns raised by individual reviewers were also addressed. As a result, the committee's overall enthusiasm for this significantly improved resubmission was very high.

DESCRIPTION (provided by applicant): Malaysia's HIV epidemic is rapidly expanding, with recent evidence suggesting increasing sexual transmission, especially in MSM. HIV prevalence among MSM is now at an all-time high of 21.6% and is concentrated in the country's capital, Kuala Lumpur, where prevalence among MSM is 43% in 2020, up from 22% just four years before. Insights into Malaysia's expanding HIV epidemic are multi-factorial: Both homosexuality and substance use are criminalized in Malaysia. MSM are, therefore, often hesitant to disclose their sexuality or risk behaviors, primarily due to fear of stigma, discrimination, or criminalization. Further, evidence suggests the widespread use (24%) of psychoactive substances (e.g., amphetamine-type stimulants) before or during a sexual encounter, also known as sexualized drug use (chemsex) among Malaysian MSM, which leads to high-risk sexual practices (e.g., condomless sex). Stigma and discrimination are also enacted on MSM by healthcare providers, which foster a hostile environment toward MSM, complicating efforts to scale-up of HIV testing and subsequent linkage to HIV prevention (pre-exposure prophylaxis) and treatment (antiretroviral therapy) services. HIV testing jumpstarts entry into the HIV prevention and treatment cascades. New HIV testing guidelines recommend MSM test every 3 to 6 months, as early HIV testing is a cost-effective strategy for stemming the HIV epidemic. Yet, HIV testing rates remain low among Malaysian MSM: ever tested (70.3%) and last year tested (30.9%). HIV self- testing (HIVST) may be particularly impactful among MSM in Malaysia. Although willingness to use HIVST is high in this group, its use is still minimal due to lack of access to HIVST kits, concerns related to misinterpreting results, and missed opportunities for counseling and linkage to care. In this context, eHealth represents an innovative platform to transform the face of HIV service delivery (i.e., testing and linkage to care). Leveraging eHealth platforms for HIV services delivery in Malaysia is ideal given that nearly all (>99%) MSM use some form of communication technology (e.g., smartphone, laptop) and has a strong preference for web-based platforms for HIVST. Therefore, we developed and pilot-tested a web-based HIVST platform, called Jom-Test®, to promote HIV testing by providing free anonymous HIVST. The results demonstrated high feasibility and acceptability for the platform but low post-test linkage to treatment and prevention services. The findings further indicated the need for real-time (online) access to counselors for pre- and post-test counseling and support for linkage to HIV and chemsex-related harm reduction services (offline). To address this evidence gap, we propose to jump-start the broader

HIV care continuum by adapting, expanding, and refining an existing Jom-Test® platform. The enhanced version, to be called Jom-TestPlus, will include real-time e-counseling (eHIVST) with integrated O2O linkage to HIV prevention and treatment services while simultaneously co-addressing chemsex-related needs for Malaysian MSM. This model represents a potentially impactful strategy for reaching marginalized populations, like MSM, and allows immediate engagement in the post-test linkage process to prevention or treatment services.

PUBLIC HEALTH RELEVANCE: The proposed research focuses on developing and testing a web-based platform, called Jom-TestPlus, that will incorporate HIV self-testing (HIVST) with real-time e-counseling (eHIVST) with online-to-offline (O2O) linkage to HIV prevention and treatment services while simultaneously co-addressing chemsex-related needs for men who have sex with men (MSM) in Malaysia. This model represents a potentially impactful strategy for reaching marginalized populations, like MSM, and allows immediate engagement in the post-test linkage process to prevention or treatment services.

CRITIQUE 1

Significance: 2
Investigator(s): 1
Innovation: 3
Approach: 3
Environment: 1

Overall Impact: This R34 application proposes to develop, implement, and evaluate an intervention that incorporates HIV self-testing, pre- and post-test counseling, and referral to care services (including HIV care). The applicants propose to adapt and refine an existing web-based platform (Jom-test) so that it is tailored for men who have sex with men who are engaged in chemsex in Malaysia. The significance of this application is high: MSM in Malaysia experience high prevalence of HIV infection, with rapidly increasing incidence rates, and chemsex engagement is associated with higher HIV risk levels; thus, there is a strong need for novel intervention approaches geared toward increasing access to HIV testing and care and counseling services imperative. While the intervention modalities are not particularly innovative on their own (HIV self-testing is not new and is being deployed using electronic modalities in many regions), holistic models geared toward chemsex participants, with accompanying supports, is novel in this setting and online-to-offline (O2O) approaches contain innovation in this context. Additional innovation added since the prior submission includes modules related to harm reduction. The investigative team is cohesive and transdisciplinary, and the core investigative team has worked together to establish the basis for this intervention via their pioneering work with Malay MSM engaged in chemsex. The research environment is excellent, with strong evidence of support from local community-based organizations working to improve health conditions among Malay MSM. The application is highly responsive to prior review comments, adding and clarifying several components including a delineation of multiple PI/PD leadership plans, heightened sensitivity thresholds for the systems usability scale (SUS), and expertise in chemsex (Dr. Lim) and addiction research (Dr. Yee). The approach proposes to a) develop the Jom-test-plus platform, pursuant to community-informed refinement and adaptation and alpha-testing; b) beta-testing the platform by which to assess usability and acceptability; and c) conducting a hybrid type-1 implementation science trial of the platform with a small number (n=78) of MSM, many of whom will have chemsex engagement experience, to assess feasibility, acceptability, and preliminary efficacy. Limitations are minor and include difficulty tracking HIV test results, a lack of attention to sex work and/or drug/sex exchange experiences, somewhat moderate methodological innovation, some lack of clarity related to operationalization of primary

outcomes (HIV testing uptake) in analyses. These weaknesses only slightly reduce enthusiasm for this generally outstanding proposal, which is considered to have high potential for impact.

1. Significance:

Strengths

- HIV prevalence among Malay MSM is high (>20%), and MSM now form the majority of new HIV cases in Malaysia.
- Applicants provide robust evidence that among Malay MSM, chemsex engagement is high and has been associated with higher HIV risk and with psychosocial health conditions that are, in turn, associated with higher HIV risk.
- The proposed intervention is based on prior work in Thailand (Adam's Love platform) and in Malaysia (Jom-test platform); however, these platforms have not previously incorporated chemsex-related intervention modules.
- Applicants have demonstrated that rates of HIV testing are low among Malay MSM; refining an electronic platform that could increase frequency of HIV testing (and subsequent care uptake for PWH) has significant potential for impact.

Weaknesses

- There remain questions about feasibility related to uptake of HIVST among Malay MSM engaged in chemsex, given low rates of verified uptake, HIV positivity rates, and subsequently confirmed care uptake rates in pilot data presented.

2. Investigator(s):

Strengths

- Strong, interdisciplinary team of investigators with expertise in implementation science, behavioral health, and epidemiology.
- Team includes founder of Adam's Love (original platform for HIVST on which this intervention has some basis; developed in Thailand).
- Team has extensive experience collaborating together.
- Core members of investigative team (Lim, Altice) have conducted pioneering research on chemsex among MSM in Malaysia.

Weaknesses

- None noted.

3. Innovation:

Strengths

- Integration into an e-platform tailored for Malay MSM involved in chemsex can be seen to constitute innovation.
- Addition e-counseling, offline access to care, and harm reduction components increase the level of innovation (and, likely, salience).

- Some additional innovation exists in how the treatment-as-usual group will be able to access many materials on the Jom-test-plus platform, except for the intervention-specific suite.

Weaknesses

- In general, the individual components of the proposed intervention are not novel in and of themselves; there is no major methodological innovation proposed, although the idea remains worthwhile and is innovative in the local context.

4. Approach:

Strengths

- The intervention approach to assess acceptability has incorporated a high threshold for system usability (>70).
- The proposed intervention development is well-grounded in a relevant theory to the target population (Behavioral Model for Vulnerable Populations).
- The Hybrid type 1 implementation science trial is presented with rigor and should allow for successful assessment of feasibility and acceptability aims (and potentially effectiveness). Notably, the “treatment-as-usual” group will receive access to the Jom-test-plus platform and its materials but be restricted from the intervention-specific modules.
- Using vouchers that can serve as tokens for free HIV testing as well as tracking uptake for this testing in the treatment-as-usual group is useful to track outcomes.
- The online-to-offline model is nicely considered; refining a workable model of this type for highly vulnerable populations to optimize linkage to care (and eventual retention) may be a hidden innovation and scalable technique if shown to be effective here.
- The intervention mapping components are nicely described and should lead to acceptable refinements to Jom-test for MSM engaged in chemsex.
- The attention given to potential moderators of intervention effectiveness is sophisticated and based nicely on prior findings.

Weaknesses

- Somewhat of a minor point, but sex work (including drug/sex exchange) is not addressed in this application and is likely at the intersection of chemsex and HIV risk for a substantial portion of MSM who may be participating in this intervention. Addressing safer sex work (perhaps in a similar module to harm reduction) might have added value here.
- Several existing e-health HIVST platforms have incorporated STI self-testing for oral, urethral, and rectal gonorrhea and chlamydia into their modalities, but there is no discussion about STI ST integration here. This is a weakness given that STI can increase propensity for HIV infection, and MSM engaged in chemsex have relatively high propensity for multiple STI.
- It is unclear why people living with HIV will be recruited in steps 1 and 2 for focus groups, rather than restricting focus groups to MSM engaged in chemsex who are HIV-negative/status unknown.
- There may be some disincentive (e.g., stigma; lack of readiness) for people who uptake HIVST and receive positive results to report these outcomes or seek help; thus, tracking such outcomes could be difficult; it may result in less missing data (especially incident HIV data) to maximally incentivize participants at 3-month and 6-month timepoints only when test results have been uploaded.

- More detail on how HIV testing frequency outcomes will be operationalized, and hypothesized effect sizes provided based on the literature, would make it easier to assess the proposal's potential to assess the efficacy aim.

5. Environment:

Strengths

- Research support environments at Yale University, the University of Malaya, and the Thai Red Cross AIDS Research Center are excellent.
- The community advisory board, though small, is already established and well-integrated in prior research and activities proposed.
- Letters of support from several local CBOs and community members show strong enthusiasm for this research proposal.

Weaknesses

- None noted.

Study Timeline:

Strengths

- The timeline is highly detailed and well thought-out, albeit for a project as ambitious as this remains somewhat compressed.

Weaknesses

- None noted by reviewer.

Protections for Human Subjects:

Acceptable Risks and/or Adequate Protections

Data and Safety Monitoring Plan (Applicable for Clinical Trials Only):

Acceptable

Inclusion Plans:

- Sex/Gender: Distribution justified scientifically
- Race/Ethnicity: Distribution justified scientifically
- For NIH-Defined Phase III trials, Plans for valid design and analysis: Not applicable
- Inclusion/Exclusion Based on Age: Distribution justified scientifically
- Inclusion and study criteria are acceptable, though some additional justification of not including participants <18 could be provided. Also - note that 18-21 year olds are generally not considered children by NIH.

Vertebrate Animals:

Not Applicable (No Vertebrate Animals)

Biohazards:

Not Applicable (No Biohazards)

Resubmission:

- The proposal is responsive to prior reviewer comments, incorporating several substantive changes that heighten its potential impact, including a stronger theoretical foundation and stronger on- and offline supports for participants.

Applications from Foreign Organizations:

- Collaborations in Malaysia are integral to this research proposal's success in the field.

Resource Sharing Plans:

Unacceptable

- Resource sharing plan was not found in this application.

Budget and Period of Support:

Recommend as Requested

CRITIQUE 2

Significance: 1
Investigator(s): 1
Innovation: 2
Approach: 2
Environment: 1

Overall Impact: This is a revised R34 with the aim of developing and testing the “Jom-Testplus” intervention to promote HIV testing and linkage to prevention and care services among 78 MSM in Malaysia randomized (1:1) to receive the intervention or treatment as usual (TAU). In addition, FGs of participants and key stakeholders will be conducted to examine implementation issues guided by the CFIR model. The revised application is highly responsive to prior critiques, and continues to be highly significant given the high rates of HIV among MSM in Malaysia that is, in part, fueled by chemsex. The investigator team and environment are very strong, and have a history of collaboration. The combination of intervention components delivered online have some innovation to them in the Malaysian context. There were a few minor weaknesses in the approach, but those did not reduce the overall importance or potential impact of the proposed work.

1. Significance:

Strengths

- Very high rates of HIV infection have been documented among MSM in Malaysia, who also face high levels of stigma, with relatively few services to address their prevention needs. The

proposed intervention addresses many of the barriers to HIV testing with its online approach and focus on promoting HIV testing and linkage to care.

- The team has documented high levels of chemsex among Malaysian MSM, which the proposed intervention addresses.
- The study team has already documented a high level of interest and uptake of HIVST on the original Jom-Test site, which suggests that the proposed adaptation may elicit similar interest and engagement given that it expands the portal to now include pre- and post-test counseling and linkage to prevention and treatment services.
- The revised application provides a stronger description of the strategies that will be used to promote online to offline services use among participants.
- Overall, the proposed work has a strong scientific premise.

Weaknesses

- None noted

2. Investigator(s):

Strengths

- PI Shrestha has extensive experience in conducting research in Malaysia and other LMICs, and has been involved in the original Jom-Test study. Dr. Shrestha is highly qualified to lead this study.
- PI Wickersham has extensive expertise in behavioral and biomedical interventions in Malaysia and other LMICs, and is an excellent choice to mPI the study.
- The PIs are joined by investigators with expertise in behavioral and biomedical interventions (including chemsex, Dr. Altice), implementation science (Dr. Altice), infectious disease (Dr. Kamarulzaman), HIV care (Dr. Raja Azwa), qualitative research (Dr. Lim), chemsex (Drs. Yee and Lim), and e-health interventions (Dr. Ng and Phiphatkhunarnon).
- The application now includes a consultant from Adam's Love (Phiphatkhunarnon)
- The MPI plan provides clear roles for PIs.

Weaknesses

- None noted

3. Innovation:

Strengths

- The combination of these intervention elements delivered online in the Malyasian context has some innovation.
- Addressing chemsex as an important target for intervention is innovative.

Weaknesses

- Video-based pre- and post-test counseling is not particularly innovative, but appropriate for the goals of the intervention.

4. Approach:

Strengths

- The proposed adaptation builds off of prior work by the study team showing strong interest and use of HIVST through the Jom-Test portal.
- The iterative development process (from FGs to alpha and beta testing prior to the pilot) is a strength of the application.
- The use of a CAB to inform intervention development is a strength, and builds off of existing relationships between the team and community partners.
- Thorough description of the qualitative analysis.
- A strength is the fidelity monitoring protocols described in the application.
- The plan to connect participants to HIV treatment or prevention services depending on the result of their HIV test is a strength.
- Strong fidelity monitoring plan.
- The revised application now describes the Adam's Love platform more thoroughly, incorporates chemsex into the intervention and the analyses, clarifies the theoretical grounding of the intervention, describes the pre- and post-test counseling more fully, sets an appropriate SUS minimum score, and eliminates an open trial of the intervention. These revisions have strengthened the proposal.

Weaknesses

- It was unclear when the chemsex elements of the intervention would be introduced.
- It is not clear why the party packs wouldn't be sent to all participants as part of the trial.
- Clarifying why focus groups would be conducted in Aim 3 as opposed to conducting individual interviews would strengthen the application.

5. Environment:

Strengths

- The revised application now includes support from Adam's Love to adapt those intervention features.
- The research environments of U Conn, Yale, the University of Malaysia, and the Thai Red Cross are excellent.
- Strong letters of support from stakeholders.

Weaknesses

- Non noted

Study Timeline:

Strengths

- Timeline ambitious but achievable with the strong study team.
- The timeline provides detailed milestones

Weaknesses

- None noted

Protections for Human Subjects:

Acceptable Risks and/or Adequate Protections

- Overall acceptable, although investigators are encouraged to provide more details on how they will train counselors to manage emotional distress due to a reactive test result.

Data and Safety Monitoring Plan (Applicable for Clinical Trials Only):

Acceptable

- Acceptable plan for identifying and managing AEs and SAEs.

Inclusion Plans:

- Sex/Gender: Distribution justified scientifically
- Race/Ethnicity: Distribution justified scientifically
- For NIH-Defined Phase III trials, Plans for valid design and analysis: Not applicable
- Inclusion/Exclusion Based on Age: Distribution not justified scientifically
- Only MSM and justified; Only Malaysian MSM and justified; children are defined as less than 18 years of age (Not 18-21), and the application does not justify excluding children less than 18 years of age.

Vertebrate Animals:

Not Applicable (No Vertebrate Animals)

Biohazards:

Not Applicable (No Biohazards)

Resubmission:

Revision:

- Highly responsive to prior critiques.

Applications from Foreign Organizations:

- Comprehensive justification for the conduct of the study in Malaysia.

Resource Sharing Plans:

Acceptable

Budget and Period of Support:

Recommend as Requested

CRITIQUE 3

Significance: 2
Investigator(s): 2
Innovation: 3
Approach: 2
Environment: 1

Overall Impact: This is a resubmitted R34 application proposing to adapt and pilot test an intervention to promote HIVST kit use and linkage to HIV prevention and treatment among men who have sex with men (MSM) in Malaysia. The investigators propose to adapt their existing eHealth intervention (Jom-Test) to include an interactive online counseling component (Jom-Test Plus) and then use a hybrid type 1 implementation design to pilot-test the adapted intervention with 78 Malaysian MSM, randomized to the intervention or treatment as usual. This project seems highly significant given the high HIV prevalence among Malaysian MSM, their low rates of regular HIV testing, and the emerging epidemic of sexualized drug use. The environment and large investigative team are excellent, with extensive track records of working collaboratively with this and similar vulnerable populations in Malaysia, and highly relevant preliminary studies. This resubmission has been largely responsive to the prior critiques. In particular, the increased attention to counseling and resources for substance use in the intervention itself and in the evaluation is a significant improvement. The behavioral conceptual model has been strengthened and is reflected in the study measurements. More information has been added about the Adam's Love intervention that will be adapted, and one of the developers of that intervention from Thailand is now included as a consultant. The design of qualitative data collection has been strengthened. Based on the prior noted strengths and these revisions, this project is likely to have high impact in the field.

1. Significance:

Strengths

- This project is highly significant given the high HIV prevalence in highly stigmatized Malaysian MSM (22% nationally, 43% in Kuala Lampur) and the low rates of regular HIV testing in this population. This is combined with an emerging epidemic of sexualized drug use (chemsex).
- The premise that an online-to-offline (O2O) eHealth intervention for HIVST will be feasible and appropriate is supported by data on the high use of smart phones by Malaysian MSM and their preferences for eHealth intervention delivery, as well as the acceptability and feasibility data from the prior version of Jom-Test.
- The team's qualitative research suggests that online access to counselors (private and confidential) plus offline support for linkage (O2O) will be more effective in increasing linkage to PrEP or HIV care than the prior iteration of Jom-Test; and could also facilitate linkage to other harm reduction support services.
- This O2O approach has also been used successfully in other countries (Thailand, Italy, Canada, and the UA).
- Collaboration with and support from the Malaysian AIDS Council and local NGOs provides promise for future implementation, sustainability, and scale-up, should the adapted tool prove to be successful.

Weaknesses

- None noted.

2. Investigator(s):

Strengths

- The investigative team has impressive expertise in public health research, infectious diseases, addiction medicine, epidemiology, qualitative and mixed methods research, behavior, intervention development and adaptation, bioethics, mHealth, HIV and substance use.
- In response to prior critiques, consultants have been added with experience with the specific intervention to be adapted and addiction psychiatry. This application adds Dr. Phanuphak, who developed and implemented a similar platform in Thailand with the NGO Adam's Love, as well as Dr. Yee, an addiction psychiatrist.
- Shrestha, the contact PI, and Wickersham, MPI, are productive junior investigators with a K01 and R21 grants as PI. Their separate and complementary roles have now been more clearly delineated in the MPI plan.
- Altice is a highly experienced senior researcher on the study topics, who also brings qualitative and mixed methods research expertise to the team. His expertise in behavioral science, and with the conceptual framework being used for the study, has been clarified.
- Dr. Karamulzaman brings senior level HIV research expertise in Malaysia and connection with local CBOs. Dr. Azwa and Dr. Lim bring research and clinical expertise with Malaysian MSM.
- Dr. Ng is a leader in eHealth.
- The investigators have a longstanding collaboration on research in Malaysia on HIV and mHealth approaches.
- The investigators have a record of collaboration demonstrated in research projects and publications.

Weaknesses

- Although one investigator has been dropped, the investigative team remains very large (even larger now) with some overlapping expertise.

3. Innovation:

Strengths

- Effective Methods to rapidly link HIVST users with prevention and treatment services are still lacking globally. This project will develop and test an innovative solution to this problem.
- Addressing chemsex along with HIV risk in the Malaysian MSM setting appears to be novel.

Weaknesses

- Some points of innovation noted (implementation science study design, use of an adaptation framework) do not seem particularly innovative.

4. Approach:

Strengths

- The approach is based on a strong and extensive record of preliminary studies by the team.
- The team already works with a CAB, and they will be highly involved in the study.

- The study is grounded in the Behavioral Model for Vulnerable Populations (BMVP) and constructs from this model will be measured at all assessment points.
- The intervention adaptation process is described in detail and will use an intervention mapping model.
- The sampling for FGs planned for in Aim 1 has been appropriately increased to capture the variation in MSM in Malaysia, and to include clinical providers as well. MSM FGs will be stratified by key variables (e.g., HIV status and chemsex use). Rationale has been provided for including HIV+ MSM and clinical providers in this part of the research.
- Intervention content and features to address chemsex and harm reduction (screening, e-counseling, harm reduction kit, linkage to resources) are integrated into the intervention and have been described in detail.
- The Hybrid Type 1 Trial seems well designed and rigorous, with good attention to intervention fidelity.
- Pilot trial primary outcomes are identified appropriately as acceptability and feasibility, with preliminary effectiveness being secondary.
- CFIR constructs will be explored qualitatively as a part of Aim 3.

Weaknesses

- It is confusing that there are sub-aims within each phase of the study that are different from the organization of the aims on the specific aims page.
- The figure illustrating Aim 1 and 2 activities mentions NGT (nominal group technique) assessment, which does not seem to be described in the methods.
- Given the centrality of substance use to the intervention, it is surprising that substance use stigma will not be measured (only HIV-related stigma).
- Focus group methods for Phase III, Aim 2 exploring implementation factors (CFIR constructs) are under-described.

5. Environment:

Strengths

- InCHIP at UConn and Yale University have strong scientific resources for this project, including several Centers and Cores to support research. Specific centers and initiatives focusing on sexual and gender minorities are described at both universities.
- The University of Malaya has a Centre for Excellence for Research in AIDS, and several other relevant centers. The Centre has experience with research with MSM and people who use drugs.
- Multiple letters of support from Malaysian organizations and NGOs--as well as the Thai team that developed an O2O model with Adam’s Love in Thailand--are included in the proposal, indicating strong local and regional support for this work.

Weaknesses

- None noted.

Study Timeline:

Strengths

- The study timeline is detailed for each phase of the study.
- The timeline and the sample size have been adjusted making recruitment plans seem more feasible.
- Recruitment and retention plans seem acceptable and feasible, given the team's experience with recruiting Malaysian MSM.
- The same retention procedures will be used in both arms of the study.

Weaknesses

- None noted.

Protections for Human Subjects:

Acceptable Risks and/or Adequate Protections

- The study team is experienced in working with this vulnerable population and has described risks and protections in detail.
- Additional information has been added as to how the team will support participants who experience psychological distress related to HIV testing or research procedures.
- A large quantity of extra information about study methods and research strategy are included in this section, which seems a bit inappropriate.
- A contingency plan for COVID-19 is included.

Data and Safety Monitoring Plan (Applicable for Clinical Trials Only):

Acceptable

- Sufficient.

Inclusion Plans:

- Sex/Gender: Distribution justified scientifically
- Race/Ethnicity: Distribution justified scientifically
- For NIH-Defined Phase III trials, Plans for valid design and analysis: Not applicable
- Inclusion/Exclusion Based on Age: Distribution not justified scientifically
- The investigators did not give a rationale for not including participants under 18 years of age.

Vertebrate Animals:

Not Applicable (No Vertebrate Animals)

Biohazards:

Not Applicable (No Biohazards)

Resubmission:

- The investigators have been largely responsive to the critiques of the prior review.

- The behavioral conceptual framework has been modified to an appropriate framework and study assessments will measure relevant constructs.
- More information on the Adam's Love intervention is included and a consultant from the group that developed this intervention has been added.
- Considerably more intervention content and evaluation design has been included for the chemsex component.
- The focus group methods have been strengthened to capture more variety in the Malaysian MSM population, and to justify the inclusion of particular groups. Qualitative analytical methods have also been clarified.
- Pre- and post-test counseling have been thoroughly described.
- No justification was provided for excluding participants under the age of 18.
- The investigative team is still large with some overlapping expertise.

Applications from Foreign Organizations:

Not Applicable (No Foreign Organizations)

- The prime organization on the application is the University of Connecticut. There is ample justification for conducing the research in Malaysia, where there is very high prevalence of HIV among MSM.

Resource Sharing Plans:

Acceptable

Budget and Period of Support:

Recommend as Requested

THE FOLLOWING SECTIONS WERE PREPARED BY THE SCIENTIFIC REVIEW OFFICER TO SUMMARIZE THE OUTCOME OF DISCUSSIONS OF THE REVIEW COMMITTEE, OR REVIEWERS' WRITTEN CRITIQUES, ON THE FOLLOWING ISSUES:

PROTECTION OF HUMAN SUBJECTS: ACCEPTABLE

INCLUSION OF WOMEN PLAN: ACCEPTABLE

INCLUSION OF MINORITIES PLAN: ACCEPTABLE

INCLUSION ACROSS THE LIFESPAN: ACCEPTABLE

COMMITTEE BUDGET RECOMMENDATIONS: The budget was recommended as requested.

Footnotes for 1 R34 MH130233-01A1; PI Name: Shrestha, Roman

+ Derived from the range of percentile values calculated for the study section that reviewed this application.

NIH has modified its policy regarding the receipt of resubmissions (amended applications).See Guide Notice NOT-OD-18-197 at https://grants.nih.gov/grants/guide/notice-files/NOT-OD-18-197.html. The impact/priority score is calculated after discussion of an application by averaging the overall scores (1-9) given by all voting reviewers on the committee and multiplying by 10. The criterion scores are submitted prior to the meeting by the individual reviewers assigned to an application, and are not discussed specifically at the review meeting or calculated into the overall impact score. Some applications also receive a percentile ranking. For details on the review process, see http://grants.nih.gov/grants/peer_review_process.htm#scoring.
